# An exploration of Irish nutrition educators’ experiences of competency-based assessment in nutrition science education

**DOI:** 10.1186/s40795-024-00906-1

**Published:** 2024-07-15

**Authors:** Sarah O’Donovan, Claire Palermo, Lisa Ryan

**Affiliations:** 1 Department of Sport, Exercise and Nutrition, Atlantic Technological University (ATU), Galway, Ireland; 2 Department of Nutrition, Dietetics and Food, Monash University, Melbourne, Australia

**Keywords:** Nutrition education, Competency-based assessment, Educator perspective

## Abstract

**Background:**

Competency-based assessment (CBA) supports the development and attainment of skills required for the workforce. Little is known about educators’ experience in developing or implementing CBA in nutrition science education or their opinions on how well it captures a student’s preparedness for the workforce. The objective of this study was to explore educators’ experience of CBA in nutrition education in Ireland.

**Method:**

Grounded in interpretivism, in-depth, semi-structured, audio-recorded interviews were conducted with 13 educators from five of the ten undergraduate honours degree nutrition programmes across Ireland. Interviews explored experiences of CBA and perception of students training to prepare for the workforce. A reflexive thematic analysis approach was implemented whereby the data were transcribed, inductively coded, and themes identified.

**Results:**

A clear divide was evident between participants who were confident in their understanding of CBA and those who were unsure or had no knowledge of the term. Those with a clear understanding were more involved in programme development and evaluation. Three themes were identified: ‘Assessment process’ including intended learning outcomes, assessment design, and grading systems, ‘Student-centred approach to assessment’ focusing on work-based assessment and preparation for the workforce, and ‘Upskilling educators’ to equip educators with the skills and knowledge for professional development and to foster student success.

**Conclusion:**

The importance of CBA as a student-centred approach, supporting them to meet standards expected to practice as a nutrition professional, was the key experience of educators. Training in CBA and inclusion of more authentic assessment may better prepare students for the workforce.

## Introduction

Registered nutritionists and dietitians are in the greatest position to help reduce the burden of non-communicable diseases (NCD) on healthcare systems worldwide [11, 28]. Dietitians predominantly through patientcare, and nutritionists through their work with individuals and the general public, work to maintain health through food and diet by providing evidence-based information on healthy eating [2, 8]. Therefore, it is necessary to ensure that a suitably qualified nutrition workforce is created to help reduce the burden of NCDs. In response to this workforce need, there has been an escalation in the number of both nutrition and dietetics degree programmes available to students entering undergraduate and postgraduate degrees worldwide. The 2019 annual report from the Association for Nutrition (AfN), an independent regulator of nutritionists in the UK, saw a steady increase in nutrition degree programmes seeking degree accreditation over a 10-year period [4].

Competency standards describe the expected attributes of professionals, guide curricula and support student development. Universally there are no compulsory professional standards for nutrition programmes on how they are developed, taught or evaluated, compared to other allied health professions whose courses and graduates are regulated by professional bodies. Competency standards for nutritionists inform professional standards for a nutrition program by outlining the knowledge, skills and attitudes a nutritionist is expected to have attained before they enter the workforce and begin professional practice [13]. From a nutrition perspective there have been some competency standards established, but these vary from country to country, and little is known about how they are assessed. The AfN have set out competency standards for nutritionists and register those who meet these standards on the UK Voluntary Register for Nutritionists (UKVRN) [5]. They provide both individual and degree accreditation on a voluntary basis using a system based on core competency areas (science, food/feed chain, professional conduct, social/behaviour, and health/wellbeing [2] and set standards to educate and train nutritionists. Degree accreditation assures incoming students that the program delivers evidence-based nutrition science education aligned to the AfN’s core competencies and standards. Whilst research was carried out in Ireland, the nutrition education system is similar to that in the UK (though science-based programs in Ireland are typically 4 years (compared to 3 in the UK) with the first year traditionally covering the core sciences (biology physics, chemistry, math)), with many Irish nutrition science degrees obtaining accreditation with the AfN. Competency standards drive competency-based education and assessment.

Competency-based assessment (CBA) measures a student’s competence by analyzing their performance and achievements and comparing them to the competence standards [14]. Assessment is a key component in education that determines whether a student is making progress and can demonstrate they can perform expected tasks upon graduation within their field of study as set out in the Miller’s pyramid framework which highlights the importance of skills beyond knowledge [12, 19, 21] completed a systematic literature review to understand how CBA has been implemented and evaluated in undergraduate/postgraduate nutrition programmes internationally to date. From 7,026 studies only 6 published manuscripts specifically detailed using CBA in the preparation of nutritionists, highlighting a large gap in nutrition science education research worldwide [21]. Universities and Institutes of Technology (tertiary education specialising in applied sciences, natural science, technology and engineering) adhere to quality frameworks and international standards, however very few nutrition educators are publishing what they are doing in competency-based education – there is no research-based analysis on how they are delivering competency-based education or how competencies are being assessed. This is in stark contrast to health professions such as dietetics [15, 17, 23, 24], nursing, and medicine [10, 26].

In most countries, dietitians (and associated use of the title) are regulated by health professional accreditation, however, without legal protection for the nutritionist title the definition and scope of practice differs greatly and has led to many unqualified individuals using the nutrition/nutritionist title. This study will focus on registered nutritionists whose scope of practice, whilst undefined in many countries worldwide, in Ireland and the UK refers to a professional who works on an individual or population level providing scientific evidence-based guidance on food and healthy eating [3, 8]. In the US these professionals may be referred to as Nutrition and Dietetics Technician, Registered (NDTR) [1]. Our research aim was to explore nutrition educators use and understanding of CBA in nutrition undergraduate education in Ireland across both accredited (with the AfN) and non-accredited programmes.

## Methodology

### Participants

A convenience sample of 13 educators teaching undergraduate honours degree nutrition programmes in Universities and Institutes of Technology across Ireland were selected based on their lived experiences [27] of assessment in nutrition programmes. All participants, except for four individuals, were recruited to the study through an invitation emailed to educators listed under the 10 nutrition programmes across Ireland. The other four participants submitted their interest to participate by supplying contact details in a survey previously distributed to all students and educators in nutrition science education in Ireland. In total, 15 educators were invited to participate, of whom 13 followed up and completed the interviews.

### Procedure

In-depth, semi-structured, audio-recorded interviews were conducted online by the first author using Microsoft Teams [9] and lasted between 25 to 60 min (M = 49.70 min, SD = 10.10 min). A semi-structured interview schedule was designed based on a literature review and researchers’ personal experiences of CBA using a mix of open-ended questions, prompts and follow-up questions to encourage interviewees to reflect on and describe their experiences around CBA in as much detail as possible. The interview schedule was broad covering many aspects of assessment within nutrition science education including their definition of CBA, experience of developing and implementing CBA, and how prepared they believed students were entering into the workforce. This paper focuses on the answers from the latter half of the questions focused on assessment (Table 1).

**Table 1:** Interview schedule outlining questions asked, and importance of the topics covered

Question	Importance
What does professionalism in nutrition mean to you?	Understand personal and professional view
What knowledge, skills and attitudes must a nutritionist possess?	Assess attributes a nutritionist must possess
How do you see the nutrition profession in Ireland progressing?	Gather opinion on how the nutrition field will progress
What barriers/challenges do nutritionists face in Ireland?	Explore barriers to progression
What does accreditation mean to you?	Gather knowledge on accreditation process and regulation
Are you aware of the Association for Nutrition (AfN)?	Explore awareness of AfN or other independent regulators
What is your understanding of competency-based assessment (CBA)?	Explore awareness of competency development and assessment
Describe your experience of CBA from teaching on a nutrition course	Gather length of time in assessment and personal assessment philosophy
In your opinion, does this assessment approach demonstrate competence against the AfN standards?	Gather opinion on accuracy of assessments
Do you believe students are well prepared to enter the workforce?	Investigate perception of students’ preparation and highlight gaps in nutrition education
If you could create a system of assessment that allows you to show how you demonstrate competence what would it look like?	Gather personal assessment philosophy

This study was grounded in interpretivism whereby we aimed to co-construct meaning of experiences of CBA between the researchers and the research participants, privileging multiple perspectives and realities. Our project team consisted of 3 members a registered associate nutritionist (ANutr), and a registered nutritionist (RNutr) based in Ireland, and a registered dietitian and experienced qualitative researcher based in Australia, each bringing unique perspectives of CBA to the data. The Irish researchers X and Y have relationships with many nutrition educators in Ireland. They run the NutriPD Community of Practice [20] bringing together nutritionists across Ireland to discuss issues and advocate for the profession. Half of the participants were members of this network, therefore having knowledge of X’s and Y’s backgrounds. X conducted the interviews as the participants were not personal associates. Although the participants may have recognized her through past interactions, this may have benefitted the project by helping with recruitment and putting participants at ease discussing their experiences teaching in nutrition with an early career nutritionist. The inclusion of a dietitian in the thematic analysis adds strength to this study as our team’s overall perspective was broadened. This team have worked together on a previous study and understand each other’s perspectives and experiences. Ethical approval for the research was granted by the ATU Research Ethics Committee after a full board review of the study proposal (RSC_AC_10062020). The study was performed in accordance with the ethical standards as set out in the 1964 Helsinki Declaration.

### Data analysis

After transcription and cleaning of the interviews, the data were analysed using the thematic analysis approach outlined by Braun and Clarke [6, 7]. This is a 6-phase guide to thematic analysis beginning with Phase 1, familiarization with the data- transcribing the data from recordings, rereading the interview transcripts and noting initial ideas on the data as a whole. Phase 2 generated initial codes, highlighting meaningful text from the transcripts, collating them into a new data set relevant to the research topic and coding based on the interesting features in the data. X and Y coded the data together and debated interpretation differences until a consensus was met. Phase 3 searched for themes in the data, grouping all relevant data items relating to each potential theme. Phase 4 involved the project team meeting to evaluate potential emerging themes, and a map of the thematic analysis was produced to analyse how well the themes captured the coded extracts. Phase 5 further defined and refined these themes, identifying the specifics of each theme starting the overall analysis. We met to review the refined themes and outline the plan for Phase 6. The final phase was the complete analysis of the data extracts identified under each theme that answered the research questions.

## Results

Thirteen participants took part in the interviews. As Table 2 outlines, participants were predominantly female (77%), had a minimum of a MSc or PhD in nutritional science or food science and had professional accreditation with one or more independent regulator- Registered Nutritionist (RNutr) with the AfN, Sport and Exercise Nutrition register (SENr), Registered Dietitian (RD) with CORU in Ireland, or Food Chemist. From the 10 nutrition programmes in Ireland 5 were represented, 3 of which held AfN accreditation at the time of the study.

**Table 2:** Demographics of nutrition educators (*n* = 13) interviewed in Ireland

Gender				Participants	
Male				3	
Female				10	
**Nutrition Programmes Represented**					
Institute 1 – BSc (Hons) Public Health Nutrition				5	
Institute 2 – BSc (Hons) Public Health Nutrition				4	
Institute 3 – BSc (Hons) Nutrition and Health Science				2	
Institute 4 – BSc (Hons) Sport and Exercise				1	
Institute 5 – BSc (Hons) Human Nutrition				1	
**Nutrition Education Qualifications**					
PhD in Human Nutrition or Nutritional Science				8	
PhD in Food and Nutrition Science				1	
MSc (Hons) in Public Health; Human Nutrition and Dietetics; Nutrition, Physical Activity and Public Health; and Applied Sport and Exercise Nutrition				4	
**Professional Accreditation**					
RNutr^a^ Only	RD^b^ Only	SENr^c^ & RD	SENr & RNutr		RD & RNutr
3	3	1	3		2

^a^*RNutr* Registered Nutritionist

^b^*RD* Registered Dietitian

^c^*SENr* Sport and Exercise Nutrition Register

Three main themes were identified. These included: (1) the assessment process, (2) student-centred assessment approach, and (3) upskilling of educators as outlined in Table 3.

**Table 3:** Developed themes, subthemes, sample codes and example quotes

**Themes**		**Subthemes**	**Sample codes**	**Example quotes**
**1. Assessment process (design and development)**	1a	*Intended Learning Outcomes*	Learning outcome focused	“In our experience, that's how we develop our assessment. When we're going through kind of module development, we focus on the learning outcomes of the module, which is kind of what the module is based around and building off that you would develop your syllabus for the module. Then the assessment we map those back to our learning outcomes, so you know we're kind of audited to check that we don't have what we call like floating assessment. You shouldn't have an assessment in a module that's not actually purposeful…Then I suppose all the learning outcomes are feeding into the overall program learning outcomes which are then based on meeting the competencies required by AfN.“ (P002)
	1b			“For us at least it's how we test students on their skills in nutrition is that they align with these competencies and our learning objectives have to line up with those competencies.“ (P011)
	1c	*Assessment process*	Stage appropriateness of assessment	“I think one of the kind of challenges are the learnings for me over this time have just been to modify them so that they are stage appropriate. I think that's been kind of the hardest piece, because when I think of the phrase competency-based assessment as opposed to learning outcome-based assessment, it makes me think of students doing something or implementing something or creating something, that's kind of what the competency piece brings in for me. Your first-year students are not going to do that. Your first-year students are going to be about learning and repeating back and providing information and telling you what they know. What I suppose is the first kind of competency category there is science knowledge.“ (P002)
	1d		Clear and concise competencies	“Just a side note, it also helps when you're trying to design a program if you've got very specific competencies that have to be achieved because then it kind of just shapes everything. (P003)
	1e		Translation of theory to practice	I think ensuring from a very early stage there's a lot of practical translation…translating that to what it means in practice…showing a lot of context specific scenarios, and even things like integration of assessments into more practical ways. Not just assessing knowledge, but actually doing it in a way that is reflective of what they might do when they have graduated “ (P010)
	1f		Grading system and level of competence	“There would be core modules and core learning objectives that we have to cover that students have to meet. In terms of third level education there is a very loose adherence to whether students have passed assessment. I mean, in some courses they have to get 30%., in some courses they have to get 40%. And not just for nutrition, but we kind of do ask ourselves if somebody gets 40% in something, are they really competent? That's probably a wider issue around the way that learning objectives are written and the way that modules are tested. Is there a chance that some student by the end of the degree might not be fully competent in some of the competency? Yeah, that's possible, because they can do some compensation of marks.“ (P011)
	1g		Thresholds and standards	“We want to know that certain people are at a certain level so that they have a minimum standard…I think it gives us as educators a level of understanding of whether you know you can go in and give a lecture and you can talk and talk and talk. But do the students understand it and then can they apply it? You can assess knowledge, but it doesn't necessarily mean they're competent to do those tasks and skills, and I think having an assessment that assesses their level of skill and being able to understand whether they meet those levels of skill to be able to be signed off as competent is really important.“ (P012)
	1h		Standardisation of training	“Needs to be a standardization around training. An agreed approach on what exactly everybody doing a nutrition degree needs to be able to deliver at the end of it if you like, and that they have demonstrated this as opposed to heard about it “ (P004)
	1i		Agreement needed on suitable assessment	“I suppose we get them in practical workshops to use a lot of the population based/food based dietary guidelines and again demonstrate their application in different settings and situations you know, so they're kind of simple examples. But as I say, I think having some agreement around what exactly everybody within the nutrition degree should have coming out is where I think I mean…I mean, you know, designing the assessments is easy enough once you're clear on what you want them to demonstrate, you just have to think about it in that way.“ (P004)
	1j		Reality of assessing competencies	“What happens in practice is that competencies aren't necessarily tested one at a time. There could be three or four competencies in one piece of assessment. Or equally it might take a couple of assessments to test one competency.“ (P011)
	1j		Effect of educator’s background	“What I do notice is that that tends to happen in modules that are taught by nutritionists or dietitians more so than other sectors.“ (P010)
	1k	*Evidence*	Tangible evidence	“Something tangible that you can say look, this student has demonstrated that they can implement this or that they understand this concept. I suppose having something kind of tangible that shows how an individual competency has been met.“ (P002)
	1l		Portfolio	“One of our external examiners recommended that we essentially kind of work with students annually for them to develop a portfolio of their skills and so kind of describe and list the various things that they have learned, and you know by the time they get into 4th year, it will be quite comprehensive and they won't have to be thinking about it retrospectively.“ (P002)
	1m		Context specific translation of theory to practice	“Health promotion is a key aspect to the degree that we …From first year, second year and into fourth year, they do competency-based learning with regards to designing health promotion activities and interventions for the general public. For example, they go right from the initial stages designing an intervention, to carrying it out and evaluating it in school-based settings and also in older adults. They’re not just learning the theory, they are actually going out doing that and learning the skills of a health promotion practitioner in the process of doing that “(P005)
	1n	*Issues associated with changing assessment*	Practicalities of changing assessment	“I think that there is work to be done in terms of modifying assessments, but I don't believe that a radical overhaul is ever the answer. I think kind of slowly modifying things, and that's just kind of the way I've learned to do things as well “ (P002)
	1o		Changing role of nutrition	“I think sometimes it can be hard to kind of have that agility within your programme to constantly be meeting the new and emerging roles for nutrition “ (P003)
	1p		Accuracy assessing student’s competency levels	“We change it all the time and then staff as well become more experienced in teaching…But I'd like to think that most of the assessment we do is competency-based. That it is assessing competencies. But…how competent are they is another question “ (P011)
**2. Student-centered approach**	2a	*Preparation for the workforce*	Performing the skill	“I'm not sure there would necessarily be huge change to the actual programmes, but on the assessment side, I suppose, really capturing not just through examination of the learning but also the ability to perform and to have specific skills that would be valued in the workforce.“ (P004)
	2b		Preparation for work-placement	“The assessments are to prepare and present three nutrition workshops…to do a case study on an internationally competitive team where they have to write menus. They must make up a supplement ordering and costing and organize travel nutrition for a squad. Identify supplement provider, things like that. Then they have a practical assessment where they do hydration testing and body composition analysis and those clearly are competency based, because they're competencies that the student needs to have achieved before they move on to semester two placement module where they can consolidate them before they graduate and start work “ (P001)
	2c		Context specific assessments	practical translation and maybe developing assessments that are more job specific, that are things that they might have to do in a scenario so that then it makes sense to them, but also gives them something to talk about in interviews as well “(P010)
	2d	*Competency development*	Framing assessment to competency	“We would use it for the dietitians on placement. It would be around framing your assessments to align it with agreed and recognized competencies that you want students to have attained. It can be kind of broken down into kind of competencies you expect them to have attained by certain time points in their learning, or kind of overall competency at the end “ (P003)
	2e		Community involvement	“Setting tasks around demonstrating that may be the kind of approach or we do this thing as a group in TUD with students learning with communities. We link with community groups and students do a real live active project. It's a collaborative approach project. I really think for nutrition this is the under-tapped possibility for learning and for demonstrating proficiencies, around the whole place of nutrition and community development “ (P004)
	2f		Importance of real-life examples	“At undergraduate level we have a second-year module and an applied sports nutrition 4th year module… students split into pairs and each student has a player from the Donegal Ladies football team to work with for the season. What they're actually doing is as a case study, they do the baseline assessment at the start of the semester, and then week on week we work through different elements of preparation that along with myself and the athlete they've come up with goals each week…They have key core competencies each week that I assess them on. But the biggest one, or where a lot of the marks are on, I really believe in it, is this translation ability, so being able to communicate complex information to someone in a way they understand. They're creating this evidence-based workbook over the course of the semester and they’re assessed on core competencies. It's essentially a mini version of an accreditation process “ (P006)
	2g		Integrating all knowledge	“They also kind of forget about all they learnt in year one and two. They have a lot of lab skills and they probably don't always see that as nutrition skills and so having the skills audit to get them to literally go through each module by module and say whatever you’ve done, whatever you learned and what skills have you developed from it as well is really important.“ (P012)
	2h	*Assessment- practical*	Role play and PBL	“Role play…with credible professional people that get briefed into playing a certain role. I did try a little bit of problem-based learning. I find it very realistic if they're done well “ (P003)
	2i		Training for one-to-one consulting	“In the UK, some registered nutritionists do one-to-one with clients, for example, you know, obviously not for clinical conditions, but maybe like for weight loss. That's the type of thing I'd love to see. I'd love to see us train our students in how to do that. Quite a lot of nutritionists do that in the UK, but we don't train our students to do that, and I think if you know some of them wanted to go into private practice and that after a couple of years I think that for example would be a really good kind of skill to develop in them.“ (P013)
	2j	*Assessment- reflective*	Reflective practice key to achieving competence	“It helps students recognize their own kind of strengths and also maybe highlights other areas they still need to improve on. Just because you did something very well and you “met the competency so to speak”, there's definitely still stuff that you could learn and develop, and I think having that reflective practice allows them to do that “ (P007)
	2k		Instill good reflection practices	“I think more reflective practice would be good, and I hate reflective practice. I'm the worst. I don't do it myself half enough and I should as a nutritionist, but I don’t, and I feel like if we could try and fill that in early on then we’d instil some good practices going forward.“ (P007)
	2l		Critical reflection	“I think critical reflection needs to be included throughout because it's something that is a difficult skill initially to master, but something that once you're graduated, you're expected to do it all of the time, and it's expected to underpin all of your CPD. So, if you've never learned to do that, then we're not going to be overly good I guess in practice.“ (P010)
	2m		Continuous portfolio	“I would like the competency-based assessment form, like a portfolio almost that you probably start maybe in year one and it tracks with you as you move through the program. And I think in alongside that kind of exercise…you have like reflective practice as well, and you'll be drawing on all the different things you learn throughout the program tying it in with the required competencies “. (P003)
**3. Upskilling of educators**	3a	*Training requirement*	Need training for CBA to be effective	“I think if it's done well and if the assessors are properly trained and if the students are trained it can be really valuable in terms of shaping their learning and kind of modelling what the learning should be “ (P003)
	3b		Workload and lecturer-student ratio	“competency based it does require more input on the behalf of the lecturer, and I just wonder looking back on my own days in college, there was less of it than there is now. I think, for example some of the institutes of technology, and I know, I work in one myself, but I think they're better at doing competency-based assessments than for example, in larger universities, with larger numbers of students “ (P005)
	3c		Require guidance	“I would definitely welcome more guidance and maybe more clear structure in terms of what types of assessment can best illustrate certain competencies.“ (P010)
	3d	*Understanding of CBA*	Unclear understanding	“competency-based assessment in my understanding, is where you're assessing, for example, whether a student has the key skills that are required. For example, something that would be competence based in different nutrition programmes could be, do they have the key skills to conduct a dietary analysis or a case study “ (P005)
	3e		Lack of clarity on how CBA is implemented	“I suppose determining that students achieved you know the competencies required to practice, so that might be knowledge based, it might be practical based…like their use of nutrition assessment tools or their ability to then take that information and to apply it to a case study or to an individual. When you're looking at courses, it's having assignments and practicals that show that the competencies that have been outlined by the AfN are being met and that can be a variety of different ways. I suppose we do it through MCQs and tests, but really I from my point of view, I think that practical based and that ability to apply it to practices is what is going to be most useful for the workplace. But also ensure that the student actually understands it.“ (P010)
	3f		Lack of clarity on how CBA is implemented	“I don't think I'm using it to any great extent. Most of the time I spend my hours in the program teaching students about food and food technology. An area that is important and not exactly the central focus of the nutrition course and so yes, we do use some level of competency assessment, especially when I'm looking at practicals.“ (P008)

(P001) = Participant 1; (P002) = Participant 2; (P003) = Participant 3; (P004) = Participant 4; (P005) = Participant 5; (P006) = Participant 6; (P007) = Participant 7; (P008) = Participant 8; (P009) = Participant 9; (P010) = Participant 10; (P011) = Participant 11; (P012) = Participant 12; (P013) = Participant 13

*CBA* Competency-based assessment, *MCQ* Multiple choice questionnaire, *PBL* Problem-based learning, *AfN* Association for Nutrition

### 1 – Assessment process

Theme one was further broken down into subthemes – intended learning outcomes, assessment process design, evidence, and issues associated with changing assessment. Throughout the assessment process nutrition educators described being focused on learning outcomes and how they align with competency standards. Many participants reported using competency standards to inform and develop assessments utilised throughout their nutrition programme to ensure students are developing competencies, and that assessments are purposeful (quote 1a, Table 3). Nutrition educators reported placing high value on the ability to put theory into practice. This is a skill that all participants agreed is important for nutrition students to learn. They explained that, where possible, it was beneficial to assess this ability in a job-specific context to prepare students for real-life work scenarios. Our participants shared their experience with assessing this ability (quote 1e Table 3).

Participants explained that it was important to consider issues associated with changing assessments in current nutrition programmes. The practicalities of which included considering the effect changes will have on accurately assessing student’s competency levels, adapting to changing roles of nutritionists in society, and most importantly the impact on the development of other skills and knowledge as competencies are not tested in isolation (quote 1j, Table 3).

Other assessment items that were identified as necessary for consideration included the stage appropriateness of assessments, linking CBA to work-based learning, the grading system, thresholds and standards, and digital capabilities. Participants noted that clear competencies are very beneficial when designing a nutrition programme as they inform the education and assessment systems implemented (quote 1d, Table 3). Concise competencies outline necessary knowledge and attributes guiding programme development to ensure students are fit to practice.

However, it was mentioned that there is a need for standardisation of the assessment approach and more work is needed to reach an agreement among nutrition educators on suitable assessments. When asked about CBA, one participant stated that agreement was needed amongst educators to specify expectations of graduates’ skills and abilities (quote 1i, Table 3). When discussing CBA and how it is implemented, one of the participants stated that they believed modules taught by nutritionists or dietitians were more focused on competency development, assessment, and preparation for the workforce (quote 1k, Table 3).

### 2 – Student-centred assessment approach

The subthemes identified under theme 2 included – preparation for the workforce, competency-development, work-based assessment, practical assessment, and reflective assessment. The majority of participants described that their programmes effectively prepared students for the workforce. Many iterated that preparedness is dependent on the effort the student puts into their education- how much they interacted with the material and the opportunities presented to them to grow and learn. Some aspects brought up for consideration to better prepare students included training on reflective practice, one-to-one client consultations, job specific translation of theory into practice and an increase in community involvement.

Reflective practice was the strongest of these subthemes throughout the interviews. Many participants stated that in order to be a competent nutrition professional reflective practice is an important skill to learn, and more emphasis should be placed on it in education to instil good habits (quote 2k, Table 3). One suggestion made to encourage more reflective practice throughout a student’s nutrition science education was to implement a portfolio-based assessment to support reflective practice through his/hers/their learning journey that would be continued for the duration of the nutrition programme from year one to graduation. A portfolio with a performance reflection form would track progress throughout the years of education along with knowledge and skills learned each year (quote 2m, Table 3).

Translation of theory to practice and job-specific skills development were also common talking points in each interview. Participants were clear that it is important to develop assessments relevant to the jobs market and test students on the skills and abilities they will need to be competent in to enter the workforce (quote 2c, Table 3). Providing job-specific scenarios to assess students’ abilities to demonstrate this application of theory into practice will increase retention of related knowledge and provide talking points for job interviews upon graduation.

### 3 – Upskilling of educators

Theme 3 was further broken down into 2 subthemes – training requirement and understanding of CBA. A clear divide was observed between participants who had a clear understanding of what CBA was and those who did not. Those who had a strong understanding of CBA were more involved in the development of the nutrition programme and had contributed to the accreditation application for their programme. The participants who were unclear on what CBA is and how it was implemented in their nutrition programmes were less involved in the programme design and development. One participant understood that the laboratory practical examinations they carried out were a form of CBA however they could not pinpoint if they used any other form of CBA methodology in their modules (quote 3f, Table 3).

The need for training in best practice CBA was consistently brought up across the interviews. Primarily in reference to training for educators, but likewise for students, on how to develop, integrate and evaluate CBA—especially in relation to portfolios, critical analysis and reflective practice (quote 3c, Table 3).

## Discussion

This study aimed to explore the experience of nutrition educators use and understanding of CBA. The analysis showed that understanding of CBA differs greatly among nutrition educators’ in Ireland, with many being unclear on what it is or how to define it. Those participants struggled to describe when it was specifically implemented. One difference between those who could define it and those who struggled is involvement in the nutrition programme design and development, or application for accreditation. Those who were involved in either of those processes had more confidence in saying what CBA was and where it was used as they had been involved in making those decisions or filling out the application forms. Overall opinion of CBA was positive with many citing the importance of assessing students’ competency levels to ensure graduates leave with the skills and knowledge necessary to practice as a nutrition professional. Furthermore, nutrition educators saw value in CBA when designing nutrition programmes as the competency standards add structure to both the course programme and assessment system. This is the first study globally to highlight current practice in CBA in nutrition programmes and highlights the need for further development of the educator workforce and instruments available to support practice^(12)^.

The findings are perhaps unsurprising considering the systematic literature review by O’Donovan et al*.* [21] outlining the small number of publications available that address CBA in nutrition science education specifically [21]. In particular, a lack of empirical evidence exploring the perspectives of nutrition educators on the education and assessment of students. Comparatively, in the dietetics profession there has been an abundance of research carried out to specifically address how dietetic students are educated and assessed to determine the strengths and weaknesses of current dietetic educational processes [17],C. [23],C. [24]. A systematic review of assessment practices in dietetics education was carried out by Jamieson et al. [15] which identified 37 studies focused on the development of assessment for trainees both in work-placement and university settings. This review highlighted the vast research being done to develop better assessment instruments for use in dietetics education and called for a more programmatic approach to CBA [15]. This distinct lack in research around CBA practices for nutrition programmes would understandably result in nutrition educators having mixed or unclear understandings on CBA and its implementation in teaching and learning. Nutrition programmes can learn from these experiences in dietetics considering the need for multiple assessment instruments and the programmatic nature of their integration needed across programmes.

Most educators called for more practical assessments of knowledge and skills, which would align with Miller’s [19] framework for competency-assessment valuing assessments that assess students’ capability to function independently and evaluate their performance. Examples of those suggested included role-play, problem-based scenarios, simulations, Objective Structured Clinical Examinations (OSCE), community projects, and work-based placements. These provide students with the opportunity to experience job-specific tasks relevant to the current labour market and set realistic expectations of the types of work they may end up taking on after graduation. The use of simulations and OSCEs with standardised patients have become common place in the education of dietetics students to develop counselling skills and prepare them for work in clinical settings. Several job opportunities would benefit from competent, upskilled nutrition educators and therefore, demonstration of competency through CBA could be of importance to highlight to employers. A systematic literature review by O’Shea et.al. [22] on using simulations in dietetics education identified 14 studies which highlighted how simulations contributed to dietetic students learning and prepared them for the workforce [22]. These types of assessments link to the ‘Shows’ and ‘Does’ levels on the Miller’s Pyramid which are necessary to implement when determining competency levels—they specifically test a student’s ability to translate theory into practice [31]. There is a need for nutrition science education to include more authentic assessment in practice by bringing in work-integrated learning.

Another recommendation that was unanimous in the interviews was the need for more reflective practice in nutrition science education. As with many health-care professions, it is an important skill which all nutritionists are expected to practice, ensuring they are keeping up with their continual professional development and practicing their critical analysis skills [16, 29]. Several nutrition educators admitted to not keeping up their reflective practice or being unsure of how to do it. These educators felt that if it had been instilled in them during their education, it would be engrained in their professional practice. Reflective practice not only allows an individual to identify their strengths, it also highlights the areas in which there is need for improvement. An early systematic review by Mann, Gordon and MacLeod in 2009 identified 29 studies focused on the development and use of reflective practice in health professions education. Many of these studies were in relation to medical or nursing education. The review highlighted the potential impact reflective practice can have on students including supporting deeper learning, developing associations between theory and practice, and improved relationships among educators and students as a result of collaborative reflection [18]. An increase in reflective practice methods, such as a yearly portfolio, across nutrition programmes would be very beneficial for students and their professional development [30]. Another potential benefit could be the use of portfolios after graduation for reflection before a job interview—providing an overview of their academic career to review before an interview and remind them of the competencies they have developed.

Training for students in one-to-one consultations was recommended by one of the nutrition educators as an aspect the student population are currently missing in their education. Currently, many students experience difficulties in obtaining their first job. Establishing a consultation business and providing private one-to-one nutrition counselling for individuals has become a popular choice for nutritionists to earn income or supplement primary earnings. UK-based freelance Registered Nutritionist Aliya Porter provides client consultation training and informed us that “with the number of low paid nutrition jobs available and lack of well-paid entry level jobs, more new graduates are turning to freelance practice in the hope of securing their income. There is real danger in doing this without experience and…many new graduates also don't realise supervision is required for ANutr working with individual clients”(personal communication, 07 October 2021). Students are not currently prepared for this in traditional nutrition programmes. When considering freelance practice graduates need to be familiar with the theory of behaviour change, ethics of dealing with clients on a one-to-one basis, understanding their scope of practice as a graduate nutritionist, and regulation to ensure safety and efficacy.

The small study sample capturing 50% of nutrition programmes across Ireland may not be transferable to other education globally. However, the participants’ years of experience and richness of data obtained from this small sample, use of existing theory and literature [15, 21, 25] to guide the line of inquiry and rigorous process of data analysis strengthens the credibility and dependability of these findings. The participants’ years of experience teaching nutrition and their role in the university/institute was not collected during the study. These factors may play a big role in their perceptions and experiences related to CBA and should be considered in future research in this area.

From the analysis of the interviews, there is a clear need for nutrition educators to upskill their CBA education to better understand what it is and how it is implemented on their nutrition programmes. Also, more guidance is necessary in the development and implementation of CBA for educators to be confident in assessing students effectively. Providing this necessary training and guidance to educators will ensure that nutrition science programmes are fostering student success. The necessity for standardisation of training, and agreement on what CBA methods are suitable to assess each of the different competency standards was vocalised too. An agreed approach for assessing students in nutrition programmes would result in more highly qualified nutrition graduates entering the workforce and foster student success. This would have a positive effect on the current burden of disease and raise the profile of nutrition professionals among the medical science community and the public. This work would be best conducted as a joint effort by both the academic nutrition science programme coordinators and the AfN, with a plan to promote this development with governing bodies in higher education to promote the need for regulation within the nutrition profession. The data from this research is part of a larger ongoing study in Ireland focused on developing a framework for CBA in nutrition science education. The next phase of research will explore students’ perceptions of CBA and their preparedness for the workforce. These studies will collectively inform the future design of nutrition science programmes.

## Conclusion

Nutrition science educators perceived CBA as an important student-centred approach to support students and future graduates to meet the standards expected to practice as a nutrition professional. Training in CBA was desired by educators to further enhance their conception of CBA and the extent to which they implement it throughout their teaching and learning. To better prepare students for the workforce the inclusion of more authentic assessment in nutrition science education should be considered to bolster competency development and employability.

## Data Availability

Data is provided within the manuscript.
